# Mutation Accumulation Underpins Evolution of Lifespan Extension by Dietary Restriction

**DOI:** 10.1111/acel.70808

**Published:** 2026-11-05

**Authors:** Sara D. Irish, Annabel Kimberley, Simone Immler, Jacob Moorad, Alexei A. Maklakov

**Affiliations:** ^1^ School of Biological Sciences University of East Anglia, Norwich Research Park Norwich UK; ^2^ Institute of Ecology and Evolution, School of Biological Science University of Edinburgh Edinburgh UK

**Keywords:** ageing, dietary restriction, life‐history evolution, senescence

## Abstract

Dietary restriction (DR) extends lifespan in animals and plants, but its evolutionary causes are elusive. The prevailing explanation is that DR prolongs life by shifting resources from reproduction to somatic maintenance. To test this idea in *Caenorhabditis elegans* we developed an experimental paradigm that tricks nematodes into increasing their reproductive effort under DR via food odour cues. Although DR improved survival, experimentally increased reproduction under DR did not reduce either immediate or long‐term survival benefits, thus contradicting resource allocation theory. Our results suggest that much of the fertility suppression under DR reflects restraint from reproducing in a poor environment. We developed a model based on Hamilton's forces of selection showing that lifespan extension under DR can evolve because DR suppresses fertility, thereby directly increasing selection against mortality in that environment. Our novel analytical approach demonstrates that DR‐driven lifespan extension can evolve under a broader range of conditions than previously anticipated, such as a relaxed need for physiological or genetic trade‐offs. Instead, we show how reduced survival on plentiful food can evolve via mutation accumulation.

## Graphical abstract

Dietary restriction extends lifespan without trade‐offs between reproduction and somatic maintenance. An evolutionary model reveals that reduced fertility strengthens selection against mortality, while mutation accumulation may explain shorter lifespans under plentiful food. Experiments in *Caenorhabditis elegans* support this explanation.

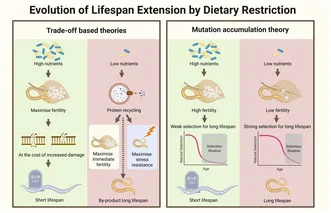

## 1 Introduction

Dietary restriction (DR), a reduction in nutrient intake without malnutrition, is well known to extend lifespan across a broad range of taxa, from plants to primates (Chmilar et al. 2024; Fontana and Partridge 2015; Nakagawa et al. 2012; Colman et al. 2014; di Francesco et al. 2024), yet the evolutionary causes of this phenomenon remain poorly understood (Schmauck‐Medina et al. 2026). There is much debate regarding the evolutionary causes of longer lifespan under reduced food intake, and whether the response to different forms of DR in the laboratory carries adaptive benefits that would translate to natural populations. DR may extend lifespan when organisms make an adaptive ‘decision’ to delay reproduction when nutrients are scarce and to allocate remaining resources to somatic maintenance until food becomes available again (the ‘resource reallocation’ theory) (Holliday 1989; Shanley and Kirkwood 2000). While this theory enjoyed some support, many empirical studies in recent decades have shown that increased lifespan and reduced reproduction under DR can be uncoupled.

Alternatively, the ‘nutrient recycling’ hypothesis suggests that lifespan extension via DR occurs through the recycling of extraneous proteins and cells, but for the purpose of maximising immediate reproduction rather than for survival. This views increased longevity as an unselected by‐product of increased recycling (Moatt et al. 2020). However, it is argued that delaying reproduction is an ineffective strategy for maximising fitness due to higher rates of extrinsic mortality in nature, especially in short‐lived organisms (Prentice 2005; Adler and Bonduriansky 2014). Finally, the ‘clean cupboards’ hypothesis argues that when nutrients are scarce, individuals will use up damaged cells and misfolded proteins to make up for the deficiency to maximise immediate survival, while improved late‐life survival is again seen as an unselected by‐product of the removal of metabolic waste (Speakman 2020). While some evidence supports both of these models (Zajitschek et al. 2023), other studies find that DR animals not only live longer than controls (Phillips and Simons 2024) but also exhibit improved health and increased tolerance to abiotic stress and pathogens (Fontana and Partridge 2015; Smith et al. 2008), and, in certain circumstances, can uncouple such benefits from fertility declines (Wei et al. 2024). This suggests that improved somatic maintenance under DR response may be beneficial for fitness under a broad range of ecologically relevant conditions.

A more parsimonious explanation for the evolution of DR is that different environments manifest different selection gradients for vital rates. Specifically, the direct effect of DR is to suppress reproduction, and this has an indirect effect of increasing selection gradients for survival, especially at late age. Provided that some genes are expressed differently in different environments, standard evolutionary theory predicts that selection will result in the evolution of longer lifespan in the environment that suppresses reproduction. The evolution of lifespan extension under DR does not require resource allocation trade‐offs but rather stems directly from the concept of ‘selection shadow’, the declining force of natural selection with advancing age (Williams 1957; Hamilton 1966), coupled with differential gene expression. Because adult survival is more important for evolutionary fitness in a DR environment compared to a non‐DR environment, the DR response—the difference in somatic maintenance between DR and non‐DR environments—can evolve via accumulation of germline mutations that reduce somatic maintenance in non‐DR environments.

Here we tested previous models for the evolution of the DR response in *Caenorhabditis elegans* nematodes using an experimental paradigm that allows us to induce reproduction in DR conditions. Environmental sensing plays an important role in the DR response (Apfeld and Kenyon 1999; Alcedo and Kenyon 2004; Libert et al. 2007), and olfactory and gustatory neurons act through partially independent pathways to influence lifespan extension (Apfeld and Kenyon 1999; Alcedo and Kenyon 2004). Previous work has shown that in *C. elegans* , introduction of the scent of food in DR conditions can partially restore reproduction at the cost of lifespan, thus supporting resource reallocation theory (Ivimey‐Cook et al. 2021). However, we developed an experimental protocol where we can substantially increase reproduction under DR by transient food removal without any effect on lifespan extension. We focused on time‐limited fasting, a form of DR where access to food is restricted over a certain period, as previous work showed that it extends lifespan (Uno et al. 2013) and improves post‐fasting reproductive performance (Ivimey‐Cook et al. 2021) relative to non‐fasting controls. Our result clearly shows that these animals have enough stored resources for reproduction under DR and suggest that suppression of reproduction is an adaptive decision to avoid producing offspring in a poor environment.

Our findings contradict resource allocation and nutrient recycling models of DR, as we further show that DR increases two measures of post‐DR fitness. These results fit the hypothesis that DR response evolves because of relaxed selection on somatic maintenance and survival in non‐DR environments (Figure 1). Because this hypothesis lacked formal theoretical foundation, we developed a new mathematical model for the evolution of the DR response via mutation accumulation (DRM) (Medawar 1952), which demonstrates that the DR response can evolve under a broad range of conditions. Physiologically, this model is close to the ‘clean cupboards’ hypothesis but instead argues that increased survival under DR is directly selected for by upregulating somatic repair and maintenance.

**FIGURE 1:**
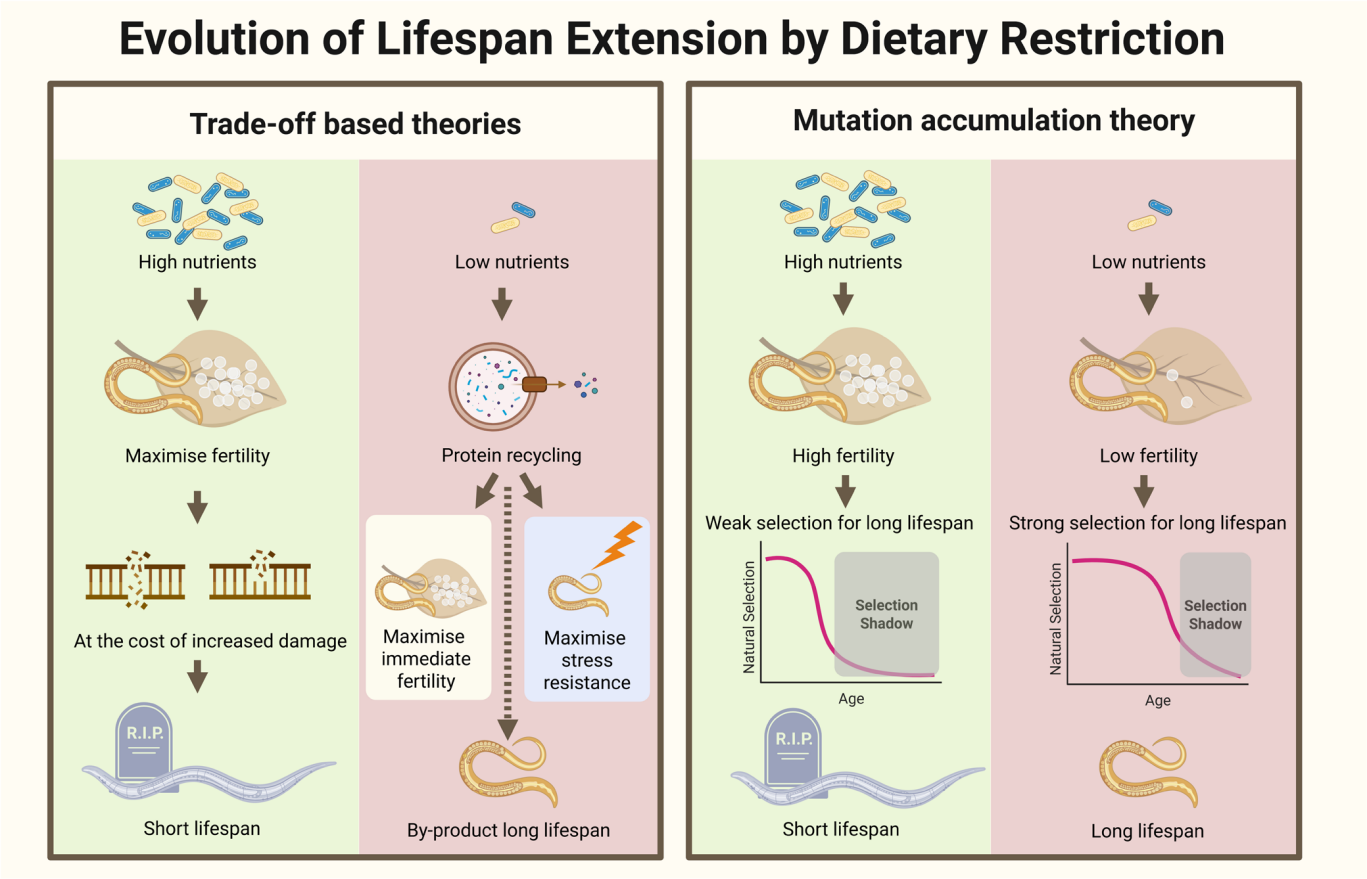
Theories for the evolution of lifespan extension by dietary restriction. Classical resource allocation trade‐off theory maintains that increased fertility comes at the cost of increased damage because fewer resources are allocated to repair when resources are plentiful. More recently, ‘recycling’ theories suggest that in low‐nutrient environments, individuals recycle extraneous proteins to either maximise immediate fertility (‘nutrient recycling’) or immediate survival (‘clean cupboards’), leading to a longer lifespan as a by‐product of toxic waste removal. Mutation accumulation theory maintains that reduced fertility under low nutrient availability, whether due to physiological resource limitations or adaptive delays in reproduction until nutrients become plentiful, leads to strong selection for longer lifespan in such environments, even in the absence of trade‐offs, resulting in the evolution of dietary restriction response. This figure was created with BioRender.com.

## 2 Results

Here we present both our empirical results from experimental work on *Caenorhabditis elegans* and our mathematical model exploring the evolution of the dietary restriction (DR) response.

In the first section, we show that delayed reproduction under DR is in part an adaptive ‘decision’ to delay offspring production until the environment improves, and that food odour‐driven increase in reproduction and growth under DR does not reduce DR lifespan.

In the second section, we present our new evolutionary model that invokes Hamilton's selection against age‐specific mortality to explain how increased lifespan can evolve in an environment where fertility is suppressed. The derivation of the model is explained, and the model conclusions are presented.

### 2.1 Empirical Results

#### 2.1.1 Food Odour Partially Restores DR‐Driven Fecundity Decrease Without Cost to Offspring

Dietary treatments significantly altered the reproductive schedule when compared to controls (Zero‐inflated negative binomial (ZI NB) GLMM: Treatment*Day^2^: *Χ*_3_^2^ = 30.688, *p* < 0.0001; Treatment*Day: *Χ*_3_^2^ = 112.655, *p* < 0.0001, Table S1). DR with food odour had an intermediate effect on reproduction (DR + O*Day^2^: *p* = 0.0003; DR + O*Day: *p* < 0.0001, Table S1), slowing it down to a lesser extent than DR alone (DR* Day^2^: *p* < 0.0001, DR*Day: *p* < 0.0001, Figure 2A). DR with or without food odour also significantly reduced lifetime reproductive success (LRS; LM: *R* ^2^ = 0.111, *F*_3,122_ = 6.219, *p* = 0.0006; DR: *p* = 0.003; DR + O: *p* = 0.017, Figure 2B and Table S2). There was some evidence that the fully fed with additional food odour treatment slightly altered the age‐specific reproduction schedule (F + O*Day^2^: *p* = 0.003; F + O*Day: *p* = 0.028, Table S1), but this does not affect our conclusions.

**FIGURE 2:**
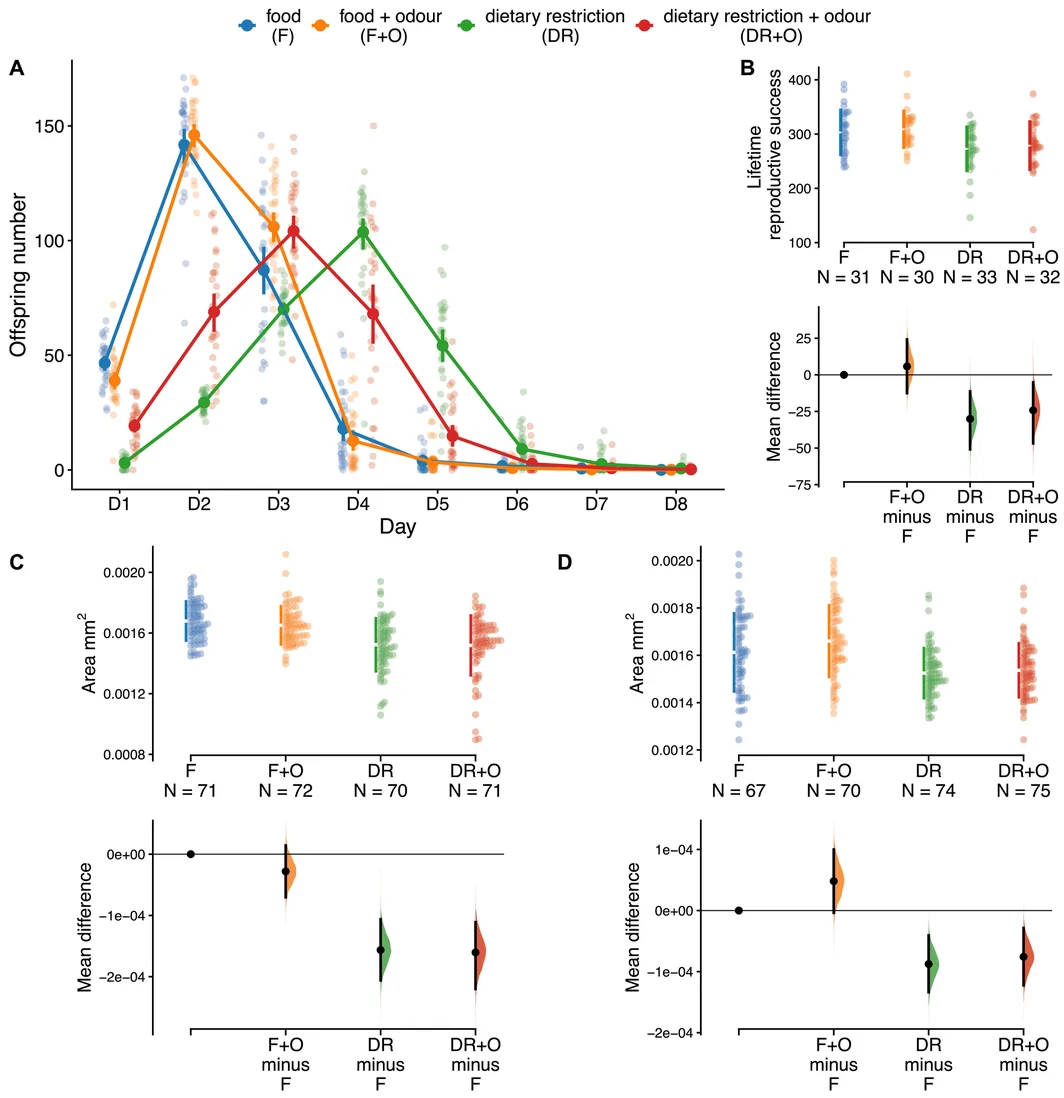
Dietary restriction slows reproduction, but to a lesser extent in the presence of food odour, without impacting egg size. (A) Age‐specific reproduction and (B) lifetime reproductive success (LRS) following treatments of *ad libitum* food (F), *ad libitum* food plus food odour (F + O), dietary restriction (DR), and dietary restriction plus food odour (DR + O). In (A) points represent mean daily offspring production with 95% confidence intervals. In (B) coloured points represent raw data with coloured lines to represent the standard deviation and the mean as the gap between these lines. Below this are bootstrapped mean differences and confidence intervals as compared to control (F) reference. Egg size (mm^2^) measured on (C) day two and (D) day four of adulthood. Coloured points represent raw data with coloured lines to represent the standard deviation and the mean as the gap between these lines. Below this are bootstrapped mean differences and confidence intervals as compared to control (F) reference.

DR with or without food odour also resulted in the production of smaller eggs than controls on Day 2 (LMM: *Χ*_3_^2^ = 27.262, *p* < 0.0001; DR: *p* < 0.001, DR + O: *p* < 0.001, Figure 2C and Table S3) and Day 4 (LMM: *Χ*_3_^2^ = 22.307, *p* < 0.0001, DR: *p* = 0.013, DR + O: *p* = 0.031, Figure 2D and Table S3).

These results suggest that under DR individuals still maintain at least partial capacity to reproduce, and if they sense that the environment is suitable, offspring can be produced with no increased cost to investment in egg provisioning.

#### 2.1.2 Dietary Restriction (DR) With or Without Food Odour Extends Lifespan

Following DR treatments both with and without food odour, lifespan was significantly extended compared to *ad libitum* controls (Cox mixed effects model (Coxme): DR: Hazard ratio = 0.350, *z* = −5.94, *p* < 0.0001; DR + O: Hazard ratio = 0.342, *z* = −6.01 *p* < 0.0001), and adding odour had no significant effect on DR‐based lifespan extension (Tukey: Est. = 0.025 ± 0.173 SE, *p* = 0.999, Figure 3 and Table S4). These results suggest that increased reproduction under DR does not affect survival.

**FIGURE 3:**
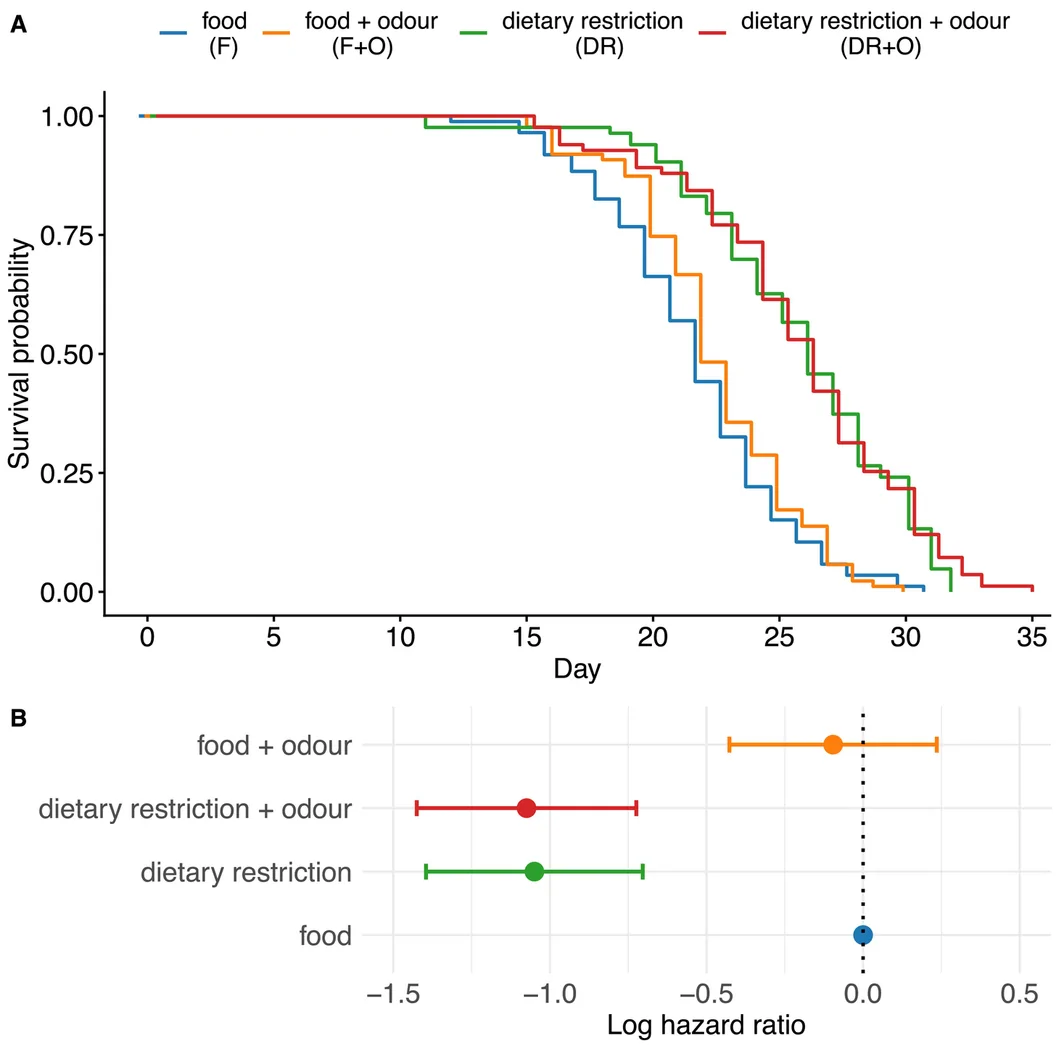
Dietary restriction extends lifespan with or without the presence of food odour. (A) Survival probability and (B) corresponding log hazard ratios following treatments of *ad libitum* food (F, *n* = 90), *ad libitum* food plus food odour (F + O, *n* = 89), dietary restriction (DR, *n* = 87), and dietary restriction plus food odour (DR + O, *n* = 87). In (B), grey dotted lines represent baseline log hazard ratio for F controls. Log hazard ratios represented by means and 95% confidence intervals.

#### 2.1.3 Mating Restores Lifetime Reproductive Success Under DR With Food Odour Exposure

Mated reproduction did not change the trends observed in age‐specific reproduction observed in unmated hermaphrodites following dietary treatment (ZI NB GLMM: Treatment*Day: *Χ*_3_^2^ = 68.701, *p* < 0.0001; Treatment*Day^2^: *Χ*_3_^2^ = 45.304, *p* < 0.0001; Figure 4A and Table S5). However, mating did allow lifetime reproductive success (LRS) to return to control levels in individuals under DR with food odour (LM: *R* ^2^ = 0.176, *F*_3,67_ = 5.997, *p* = 0.001; DR + O: *p* = 0.565), but not for those treated with DR only (*p* = 0.002, Table S6 and Figure 4B). The restoration of total reproductive output through mating under DR with food odour suggests that DR individuals can produce the same number of oocytes as fully fed individuals when access to sperm is unlimited.

**FIGURE 4:**
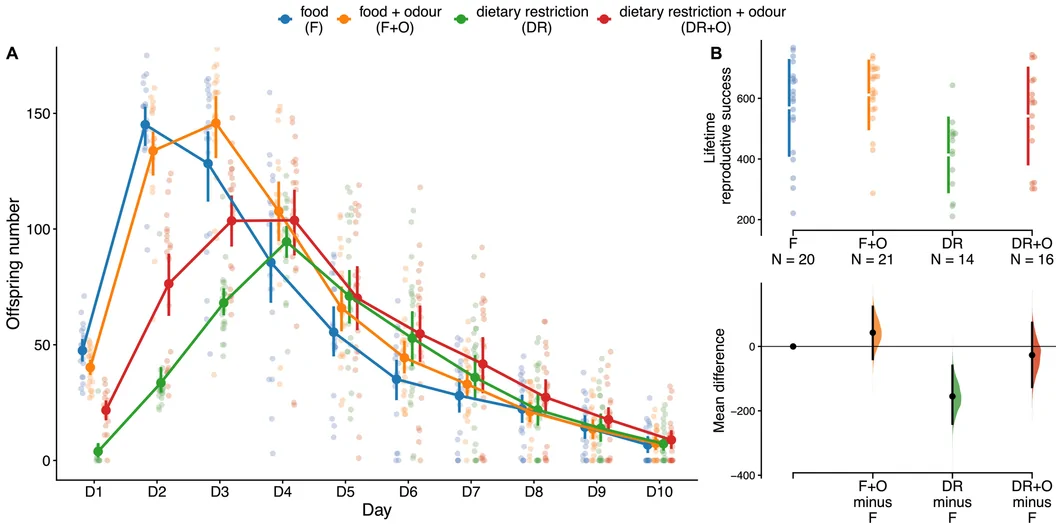
Mating restores lifetime reproductive success and partially restores individual fitness under dietary restriction with the presence of food odour. (A) Mated age‐specific reproduction and (B) lifetime reproduction success (LRS) following treatments of *ad libitum* food (F), *ad libitum* food plus food odour (F + O), dietary restriction (DR), and dietary restriction plus food odour (DR + O). In (A) points represent mean daily offspring production with 95% confidence intervals. In (B) coloured points represent raw data with coloured lines to represent the standard deviation and the mean as the gap between these lines. Below this are bootstrapped mean differences and confidence intervals as compared to control (F) reference.

#### 2.1.4 Food Odour Under DR Increases Growth Rates

DR with or without food odour resulted in smaller body size compared to controls on Days 2 (LM–*R* ^2^ = 0.929, *F*_3,92_ = 415, *p <* 0.0001; DR: *p* < 0.0001; DR + O: *p* < 0.0001) and 4 (LM: *R* ^2^ = 0.744, *F*_3,92_ = 92.77, *p <* 0.0001; DR: *p* < 0.0001; DR + O: *p* < 0.0001, Figure 5 and Table S7). However, DR treated individuals with or without food odour grew more than controls between Days 2 and 4 (LM: *R* ^2^ = 0.503, *F*_3,92_ = 33, *p <* 0.0001; DR: *p* < 0.0001; DR + O: *p* < 0.0001, Figure 5 and Table S7), and DR with food odour induced faster growth than DR alone (Tukey—Est. = −0.004 ± 0.001 SE, *p* = 0.031, Table S8). This faster growth rate with the introduction of food odour suggests that inducing reproduction does not carry costs to growth. In fact, this result indicates that animals under DR carry enough resources to grow faster, but do not do so.

**FIGURE 5:**
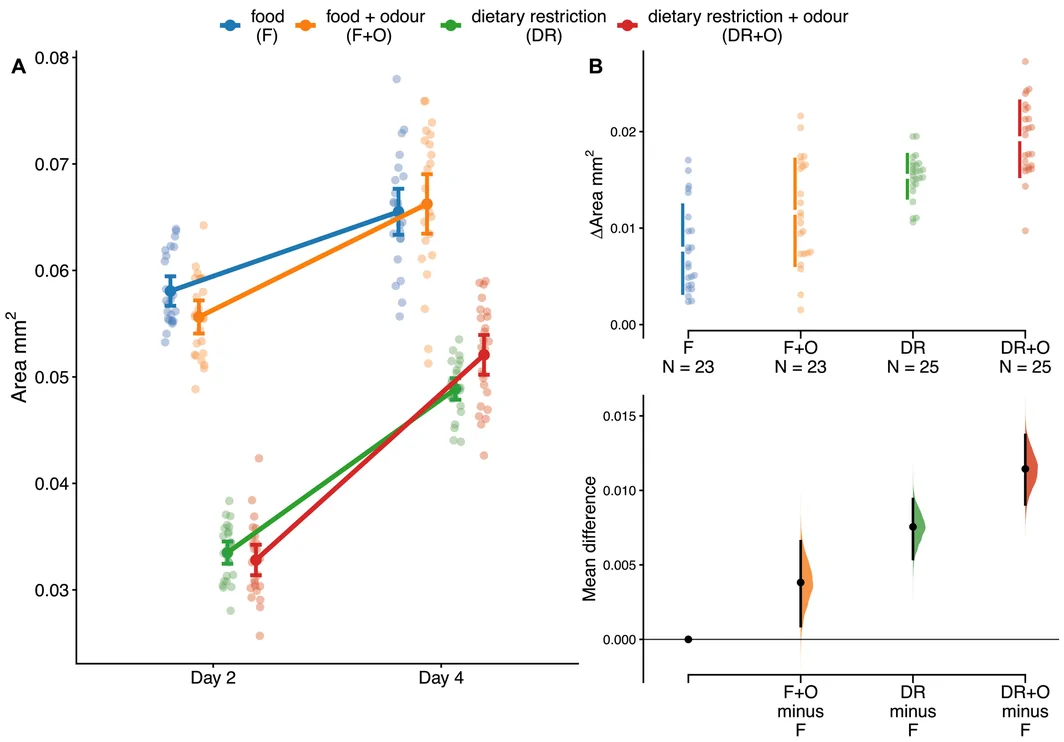
Presence of food odour under dietary restriction increases growth rates. (A) Body size measured as surface area (mm^2^) on days 2 and 4 of adulthood and (B) growth rates (change in body surface area between days 2 and 4 of adulthood). (A) Coloured points represent mean body size with 95% confidence intervals and transparent points represent raw data. (B) Coloured points represent raw data with coloured lines representing the standard deviation and the mean in the gap between these lines. Below this are bootstrapped mean differences and confidence intervals as compared to control (F) reference.

#### 2.1.5 DR Improves Stress Resistance and Does Not Suppress Population Growth in Semi‐Natural Conditions

DR treated individuals with or without food odour survived longer than fully‐fed controls (F) following heat shock (Coxme—DR: Hazard ratio: 0.042 ± 0.211 se, *p* < 0.0001; DR + O: Hazard ratio: 0.032 ± 0.214 se, *p* < 0.0001, Figure 6 and Table S9), and food odour under the *ad libitum* treatment had no effect on survival (Coxme—F + O: Hazard ratio: 1.097 ± 0.144 se, *p* = 0.522, Figure 6 and Table S9). In fact, all fully fed individuals died before the end of the heat shock treatment. However, DR only and DR with food odour treated individuals survived up to 8‐ and 12‐days post‐heat shock, respectively, suggesting not only that DR improves stress resistance, but also that allocating resources to reproduction under DR does not incur costs to early‐life survival in stressful conditions.

**FIGURE 6:**
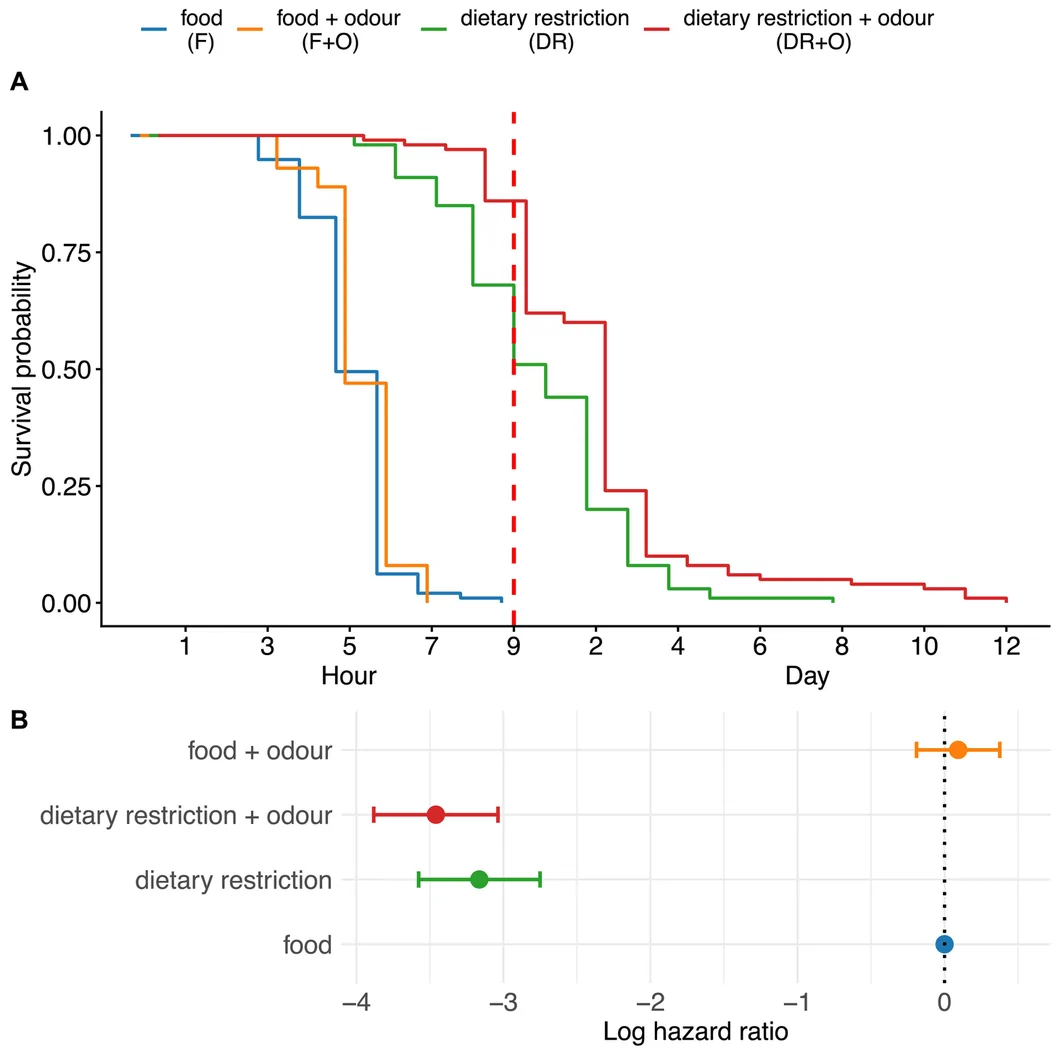
Dietary restriction with or without the presence of food odour improves resistance to heat stress. (A) Survival probability and (B) corresponding log hazard ratios following treatments of *ad libitum* food (F, *n* = 97), *ad libitum* food plus food odour (F + O, *n* = 100), dietary restriction (DR, *n* = 100), and dietary restriction plus food odour (DR + O, *n* = 100). The red dotted line represents the final hour of heat shock and the last hourly check for survival. Data to the right of this line were obtained through daily mortality checks following the heat shock. The grey dotted line represents baseline log hazard ratio for F controls. Log hazard ratios represented by means and 95% confidence intervals.

Our results did not indicate any cost to population growth following DR compared to fully fed controls. They point to a strong influence of environment on treatments, however, as is indicated by significant interactions with Block for all treatments across days and the quadratic variable for day (ZI NB GLMM: Treatment*Day: *Χ*_6_^2^ = 48.332, *p* < 0.0001, Treatment*Day^2^: *Χ*_6_^2^ = 40.668, *p* < 0.0001 Tables S10 and S11; Figure 7). Populations treated with DR and food odour did not incur substantial costs to population growth in semi‐natural conditions. This result suggests that improved lifespan, stress resistance, and growth following induced reproduction under DR are unlikely to be solely a laboratory artefact.

**FIGURE 7:**
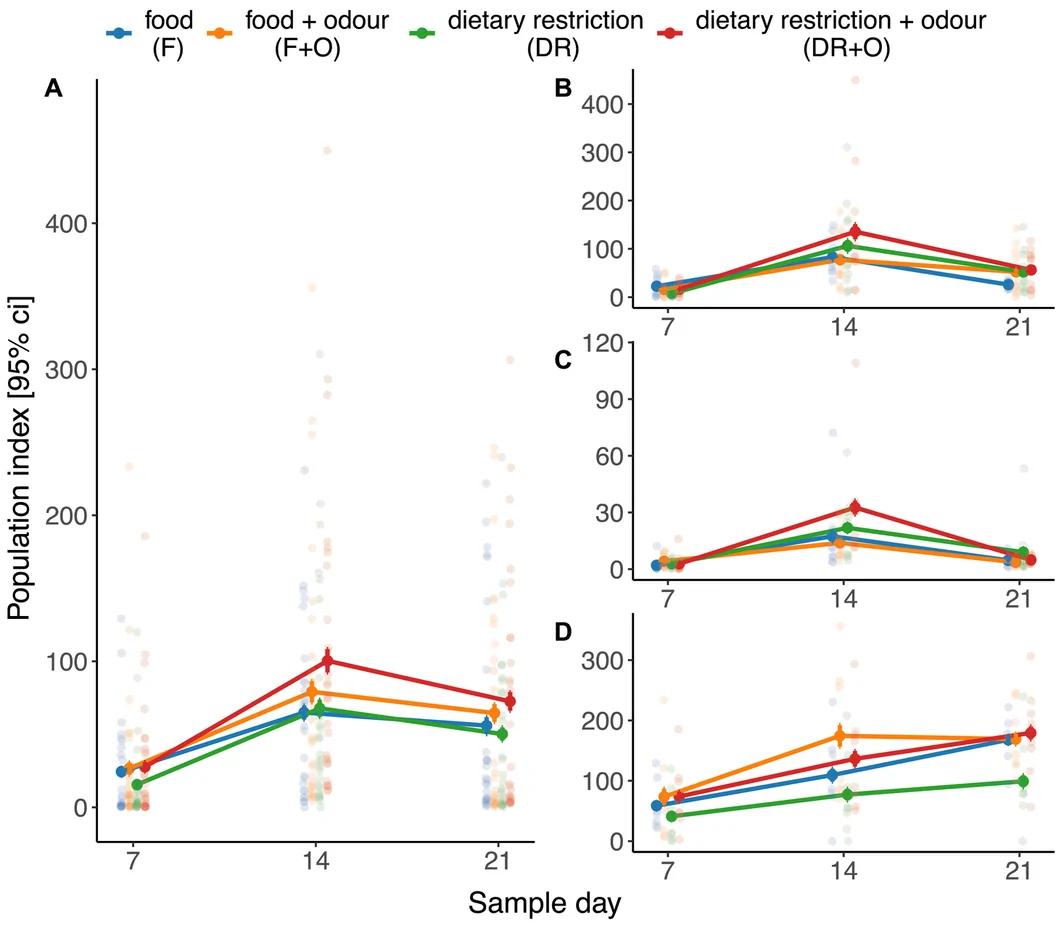
Early‐life dietary restriction (DR) does not suppress post‐DR population growth in semi‐natural conditions. Population index, measured as the number of individuals counted in a compost sample from an outdoor microcosm population founded by 50 individuals following treatments of *ad libitum* food (F, *n* = 26), *ad libitum* food plus food odour (F + O, *n* = 27), dietary restriction (DR, *n* = 28), and dietary restriction plus food odour (DR + O, *n* = 29). (A) shows data across all blocks, and (B–D) show data from individual blocks 1, 2, and 3, respectively. Transparent points represent raw data (mean number of individuals across six technical replicates per population), dark points represent means per treatment on a given day, and error bars show 95% confidence intervals.

### 2.2 Evolutionary Model

We imagine an age‐structured population with overlapping generations that is exposed to a sequence of two different environments, $E_{1},E_{2}$, which correspond to *ad libitum* and DR conditions, respectively. We assume that a move from $E_{1}$ to $E_{2}$ decreases age‐specific fertility by proportion 1−k regardless of age. A shift from one environment to another will cause changes in the age structure that will take some time to equilibrate (stability). However, the population is free to grow or shrink while stable. The duration of each environmental exposure is assumed to be much longer than it takes to reach stability.

Fertility comes at a cost to survival, and this cost can vary with the environment. In $E_{1}$, each reproductive event increases the immediate mortality rate of the asexual parent linearly in proportion to $c_{1}$; in $E_{2}$ this is $c_{2}$. Both are non‐negative. If there is a greater survival cost of a reproductive event in the DR environment than with *ad libitum*, then $c_{2}>c_{1}$. The added mortality realised in each environment follows from this per‐unit cost and the rate of age‐and‐environment‐specific fertility. The difference between realised costs of fertility in $E_{2}$ and $E_{1}$ is therefore $\delta =kc_{2}-c_{1}$.

Finally, the population has genetic variation for age‐specific mortality at all ages, and the genes that contribute to that variation are assumed to function in a strictly age‐and‐environment specific manner (i.e., there is no genetic correlation across mortality at different ages or across different environments). Genes that are specific to one environment are exposed to the effects of natural selection only when the population experiences that environment. At other times, these genes are neutral with respect to fitness.

The model that follows describes how fertility suppression experienced under DR ($E_{2}$) shifts selection to favour slower senescence. Selection for age‐specific mortality is derived using results from Hamilton (Hamilton 1966). A between‐environment comparison of selection gradients finds conditions that favour the evolution of longer life in the fertility‐suppressed environment ($E_{2}$).

#### 2.2.1 Selection Against Age‐Specific Mortality

Selection against age‐specific mortality is expressed by Hamilton (1966) as equal to

(S1) $$s\left(\mu \left(x\right)\right)=-\int_{y=x}^{\infty }\exp\left(-\mathrm{ry}\right)L\left(y\right)m\left(y\right)\mathrm{dy}$$

where $L\left(y\right)$, $m\left(y\right)$ are age‐specific rates of cumulative survival and fertility (vital rates), and r is the Malthusian rate of population growth (Fisher 1930). Selection is expressed per generation, rather than per unit time (Charlesworth 1994) We note two important properties of (S1).

1. For all pre‐reproductive ages $x<x_{A}$, the integrand in (S1) is equal to zero because $m\left(x\right)=0$.
2. Following from #1 and the Euler‐Lotka equation, $\int_{0}^{\infty }\exp\left(-\mathrm{ry}\right)L\left(y\right)m\left(y\right)\mathrm{dy}=1$, the integral in (S1) taken over all adult ages must equal +1.

It therefore follows that any change to values $L\left(y\right)$, $m\left(y\right)$ at any age *y* or to r must cause changes to vital rates at the same or other ages and/or changes in *r*.

To model how DR could affect changes in vital rates and how these might alter patterns of natural selection, we assume that a move from $E_{1}$ to $E_{2}$ decreases age‐specific fertility by the same factor 1−k, regardless of age. Age‐specific selection in these two environments is therefore equal to

(S2a) $$\begin{array}{c} s\left(\mu \left(x|E_{1}\right)\right)=-\int_{x}^{\infty }\exp\left(-r\left(E_{1}\right)y\right)L\left(y|E_{1}\right)m\left(y|E_{1}\right)\mathrm{dy}\; \end{array}$$

and

(S2b) $$\begin{array}{c} s\left(\mu \left(x|E_{2}\right)\right)=-\int_{x}^{\infty }k\cdot \exp\left(-r\left(E_{2}\right)y\right)L\left(y|E_{2}\right)m\left(y|E_{1}\right)\mathrm{dy} \end{array}$$

Note that the fertility suppression expressed in $E_{2}$ must either increase cumulative survival or reduce the population growth rate (or both) in order to meet the mathematical requirement that the integral taken over all ages equals +1. Biologically, this can be caused by the direct reduction of population growth (which will inflate the value of the exponential term). Furthermore, if there are costs to reproduction that are immediately manifested on mortality, then a relaxation of mortality costs associated with suppressed reproduction can increase $L\left(y|E_{2}\right)$ at adult ages, and this may also inflate the integrand.

For each age and environment, the total change to the population age‐specific mortality rate caused by fertility (the realised costs) is proportional to the corresponding fertility,

(S3a) $$\begin{array}{c} ∆\mu \left(x|E_{1}\right)=c_{1}m\left(x|E_{1}\right) \end{array}$$

(S3b) $$\begin{array}{c} ∆\mu \left(x|E_{2}\right)=kc_{2}m\left(x|E_{1}\right) \end{array}$$

If we benchmark age‐specific mortality in E2 by that in E1, then the former is expressed in terms of the mortality and fertility of the latter

(S4) $$\begin{array}{c} \mu \left(x|E_{2}\right)=\mu \left(x|E_{1}\right)+\mathrm{\delta m}\left(x|E_{1}\right) \end{array}$$

where δ is the environmental difference in realised fertility costs. We assume no other ecological causes for mortality differences between the environments.

#### 2.2.2 Strategy for Determining When Fertility Suppression Enhances Selection for Adult Lifespan

In principle, one can ask directly how selection changes with age *differently* in the two environments by directly taking the ratio of (S2a) and (S2b). However, it is not clearly possible to make general theoretical predictions directly from this ratio owing to the integration functions. Nevertheless, one can make useful predictions using the ratios of the integrands that are specific to common ages. If the ratio of the integrands in (S2b) to (S2a) *increases* with age, then selection against age‐specific mortality weakens with age more slowly in $E_{2}$ than in $E_{1}$. In this case, selection will act more strongly in $E_{2}$ both to resist the evolution of actuarial senescence and to enhance adult lifespan (because selection for mortality at each adult age is stronger in $E_{2}$ than in $E_{1}$). This ratio of integrands is simply

(S5) $$\begin{array}{c} R\left(y\right)=k\cdot \exp\left(\left(r\left(E_{1}\right)-r\left(E_{2}\right)\right)y\right)\;\frac{L\left(y|E_{2}\right)}{L\left(y|E_{1}\right)} \end{array}$$

Because the model does not recognise any feature that might change either the age of reproductive onset or survival to that point, the cumulative lifespan ratio can be restated in terms of adult age‐specific mortality. Because $L\left(y\right)=\exp\left(-\int_{0}^{y}\mu \left(v\right)\mathrm{dv}\right)$, the cumulative survival ratio in (S5) is equal to $\gamma =\exp\left(-\int_{x_{A}}^{y}\left(\mu \left(v|E_{2}\right)-\mu \left(v|E_{1}\right)\right)\mathrm{dv}\right)$. We can incorporate costs of reproduction into the ratio of cumulative survival by substituting (S4) into this, yielding

(S6) $$\begin{array}{c} \gamma =\exp\left(-\delta \int_{x_{A}}^{y}m\left(v|E_{1}\right)\mathrm{dv}\right)\; \end{array}$$

Following the logic described above, we can infer that selection for adult lifespan is always stronger in $E_{2}$ than in $E_{1}$ if the ratio given in (S5) has a non‐decreasing monotonic relationship with *y*. The conditions sufficient to satisfy this requirement correspond to those that find the first derivative of $R\left(y\right)$ taken with respect to *y* to be always greater than zero.

#### 2.2.3 Deriving the Conditions That Enhance Selection for Adult Lifespan

Following this logic for all values of *y*, the critical values are given by the inequality

(S7) $$\begin{array}{c} k\cdot \exp\left(\left(r\left(E_{1}\right)-r\left(E_{2}\right)\right)y\right)\;\left(\frac{\mathrm{d\gamma}}{\mathrm{dy}}+\left(r\left(E_{1}\right)-r\left(E_{2}\right)\right)\gamma \right)>0 \end{array}$$

where from (S6), $\frac{\mathrm{d\gamma}}{\mathrm{dy}}=-\mathrm{\gamma \delta m}\left(y|E_{1}\right)$. Substituting into (S7), this is

(S8) $$\begin{array}{c} \mathrm{\gamma k}\cdot \exp\left(\left(r\left(E_{1}\right)-r\left(E_{2}\right)\right)y\right)\;\left(\left(r\left(E_{1}\right)-r\left(E_{2}\right)\right)-\mathrm{\delta m}\left(y|E_{1}\right)\right)>0 \end{array}$$

and because k,γ and $\exp\left(x\right)$ must be greater than zero for all *x*, this inequality simplifies to

(S9) $$\begin{array}{c} r\left(E_{1}\right)-r\left(E_{2}\right)>\mathrm{\delta m}\left(y|E_{1}\right) \end{array}$$

The conditions work in a simple way. Reducing the population growth rate alone (the left‐hand side of (S9)) shifts the integrand in Expression (S1) towards older ages, thereby increasing selection. This is resisted at each age by any increase in realised reproductive costs in $E_{2}$ and assisted by any decrease. When the inequality is met at some ages, but not at others, then a weak form of selection‐enhancement is predicted whereby selection against age‐specific mortality may be enhanced only at some ages. The strong form, enhanced selection at every adult age, is guaranteed when the inequality is satisfied at every adult age; this is the form that is explored from this point on. A complete analysis of these conditions requires specific vital rate schedules. Instead, we consider analytically several special cases.

#### 2.2.4 Case 1: Equal Realised Costs ($c_{1}=kc_{2}$, Including no Costs)

The condition reduces to $r\left(E_{1}\right)>r\left(E_{2}\right)$. Because cumulative survival is unchanged and fertility is suppressed in $E_{2}$, the population growth rate must decrease. The inequality is met at every adult age.

#### 2.2.5 Case 2: Age‐Independent Fertility

In the general case, mortality in environment *i* and age *x* derives from components that reflect the cost of fertility and those that do not, $\mu^{*}\left(x\right)$,

(S10) $$\begin{array}{c} \mu \left(x|E_{i}\right)=\mu^{*}\left(x\right)+c_{i}m\left(x|E_{i}\right) \end{array}$$

Both components are reflected in cumulative survival to any reproductive age *y*

(S11) $$\begin{array}{c} L\left(y|E_{i}\right)=L^{*}\left(y\right)\cdot \exp\left(-\int_{x_{A}}^{y}c_{i}m\left(v|E_{i}\right)\mathrm{dv}\right) \end{array}$$

where $L^{*}\left(y\right)$ is cumulative survival to *y* that neglects the costs of fertility.

Given our assumption of age‐independent fertility, this simplifies in each environment to

(S12a) $$\begin{array}{c} L\left(y|E_{1}\right)=L^{*}\left(y\right)\cdot \exp\left(-c_{1}m\left(E_{1}\right)\left(y-x_{A}\right)\right) \end{array}$$

and

(S12b) $$\begin{array}{c} L\left(y|E_{2}\right)=L^{*}\left(y\right)\cdot \exp\left(-kc_{2}m\left(E_{1}\right)\left(y-x_{A}\right)\right) \end{array}$$

We substitute each into Euler‐Lotka and re‐arrange,

(S13a) $$\begin{array}{c} m\cdot \exp\left(c_{1}mx_{A}\right)\int_{x_{A}}^{\infty }L^{*}\left(y\right)\cdot \exp\left(-\left(r\left(E_{1}\right)+c_{1}m\right)y\right)\mathrm{dy}=1 \end{array}$$

and

(S13b) $$\begin{array}{c} \mathrm{km}\cdot \exp\left(c_{2}\mathrm{km}x_{A}\right)\int_{x_{A}}^{\infty }L^{*}\left(y\right)\cdot \exp\left(-\left(r\left(E_{2}\right)+c_{2}\mathrm{km}\right)y\right)\mathrm{dy}=1 \end{array}$$

Returning to our general condition, under our assumption of age‐independent fertility, the condition given in Equation (S9) can be restated as,

(S14) $$\begin{array}{c} r\left(E_{1}\right)+c_{1}m>r\left(E_{2}\right)+c_{2}\mathrm{km} \end{array}$$

Each side of (S14) quantifies the environment‐specific sum of the growth rate and the cost hazard. Note that both sides of this condition correspond to the arguments of the integrals in identities given in (S13a) and (S13b). Equation (S14) is satisfied if and only if the integral in (S13a), which decreases as its argument increases, is smaller than that in (S13b). Equivalently, the prefactor of (S13a) must exceed that of (S13b). Re‐arranging that inequality yields the conditions that guarantee stronger selection in $E_{2}$ under our assumption of age‐independent fertility,

(S15) $$\begin{array}{c} \mathrm{\delta m}x_{A}<\ln\left(1/k\right) \end{array}$$

Because *k* is constrained to be less than 1 by the model, the right‐hand side of (S15) must be positive. This demonstrates that selection against age‐specific mortality is stronger in $E_{2}$ than in $E_{1}$ at all adult ages (given our assumption of age‐independent fertility) in three cases:

1. there is no pre‐reproductive period ($x_{A}=0$);
2. realised costs are no higher in $E_{2}$ (δ≤0); and
3. in limited cases when realised costs are higher in $E_{2}$, up to a limit, $\ln\left(1/k\right)/mx_{A}>\delta >0$. Stronger suppression raises the tolerance, and higher fertility and delayed onset of reproduction lower it.

#### 2.2.6 Case 3: Age‐Specific Fertility and Lower Realised Costs in $E_{2}$ (δ<0)

Under this assumption, $-\delta =\left|\delta \right|$, and (S9) is equivalent to $r\left(E_{2}\right)-r\left(E_{1}\right)<\left|\delta \right|m\left(y|E_{1}\right)$. This holds at every reproductive age if and only if it holds at the age of lowest fertility

(S16) $$\begin{array}{c} r\left(E_{2}\right)-r\left(E_{1}\right)<\left|\delta \right|\min\left(m\left(y|E_{1}\right)\right) \end{array}$$

The condition is always met when the population growth rate is reduced in $E_{2}$. It can also be met when growth is higher in $E_{2}$, but that increase can be no more than the reduction in cost hazard at the adult age of lowest fertility.

#### 2.2.7 Case 4: Age‐Specific Fertility and Higher Realised Costs in $E_{2}$ (δ>0)

In the general case, the condition in (S9) must be met at every reproducing age. The threshold growth rate difference is $\mathrm{\delta m}\left(y|E_{1}\right)$. This threshold is positive, given our assumption of higher realised costs, and is most stringent at the age of maximal fertility. Therefore, (S9) is satisfied at every adult age if and only if

(S17) $$\begin{array}{c} r\left(E_{1}\right)-r\left(E_{2}\right)>\delta \max\left(m\left(y|E_{1}\right)\right) \end{array}$$

The reduction in growth rate must exceed the increase in cost hazard at the age of highest fertility.

#### 2.2.8 Evolution of a Plastic Lifespan Response

We have made two main model assumptions. The first involves genetic architecture (age‐and‐environment‐specific gene action), and the second involves environmental and physiological conditions (fertility suppression and costs to reproduction). When combined, our model predicts the evolution of among‐environment variation in adult lifespan and actuarial senescence between environments that represent *ad libitum* and DR conditions; this is an evolved plastic response. The predictions apply to a broad range of parameter values. Perhaps the most compelling is the prediction that fertility suppression by itself (Case 1) is sufficient to drive the evolution of divergent lifespan. This happens because the population growth rates are suppressed under DR, and this change alone enhances selection against age‐specific mortality by shifting the age structure of parents towards older individuals.

When mortality costs of reproduction are added to the model, the predictions become more complex because costs also contribute to shifts in the age structure. Even so, clear predictions emerge. If age‐specific fertility is held constant over adult ages, then suppression leads to the evolution of lifespan extension in the DR environment under a variety of conditions, including when there are no pre‐reproductive ages or when realised costs are no higher under DR than under *ad libitum* conditions (Case 2). When we allow fertility to vary across ages, lifespan extension is still expected to evolve when the realised mortality costs are lower in the DR environment, provided that the population growth rate is reduced (Case 3). It is possible that fertility suppression with lowered costs in the DR environment can lead to increased growth rates if the effects of enhanced survival outweigh those caused by reduced fertility, but prolonged life will still evolve if the increase in growth rate is smaller than the reduction in cost hazard at the adult age of least reproduction (Case 3). Finally, selection for long life can be enhanced under DR even if the realised mortality costs of reproduction are greater, but this requires that the growth rate shrinks by more than the mortality costs increase at the most fertile age (Case 4).

#### 2.2.9 Population Stability

The model of selection provided by Hamilton (1966) assumes stable age distributions. As noted above, this requires that the durations of each environment are long. This feature has positive and negative attributes. On the one hand, this reveals that the DR lifespan response can evolve when individuals never experience more than one environment. In fact, the greater the proportion of individuals that *do not* experience both environments, the better this assumption is met. This prediction is novel, as other models imply that the selection that leads to the evolution of the DR response is driven by individuals waiting out bad times in hopes of living long enough to experience good times. On the other hand, our model cannot cope directly with environmental fluctuations that are rapid (when compared to the lives of the individuals). However, we can describe how the models break down and make a reasonable inference about evolutionary outcomes in these cases.

The evolution of the plastic DR response described in our models is predicated on divergent selection regimes that are driven directly by differences in the age structure of new parents (the integrand in (S1) defines the probability distribution function of these parents). These structural differences emerge over time as the age structure equilibrates after an environmental shift; it is only at the demographic equilibria where Hamilton's expressions are strictly correct. However, it is reasonable to expect that as the age‐structure shifts from the equilibrium state at one environment to that of another, selection shifts from the one deriving from the first equilibrium to the one deriving from the second. While selection is in flux, it is likely to reflect a character that is intermediate to selection regimes defined at both equilibrium states. It is also reasonable to expect that as the duration of each environment shortens, this intermediate selection regime operates at a greater proportion of time. If selection becomes more intermediate over time, then it must follow that the magnitude of the plastic lifespan response will evolve to be less and less. Differently put, the potential for this mechanism to drive the evolution of the DR response follows inversely to the frequency of environmental shifts.

The model that we described proposes an evolutionary pathway to extended lifespan under DR conditions that operates in a fundamentally different way and under different conditions than those previously proposed. In addition, it is the first formal evolutionary model of the phenomenon that leverages Hamilton's description of selection gradients, a cornerstone of the modern evolutionary theory of ageing (Charlesworth 1994; Rose 1991; Charlesworth 2000).

## 3 Discussion

The evolution of lifespan extension under dietary restriction (DR) has been thought either to be driven by reallocation of resources from reproduction to somatic maintenance, or as a by‐product of nutrient recycling. Our empirical results challenge both suppositions. We show that DR animals can maintain stored resources that would allow them to increase their reproductive rate, up to a point, without any effect on lifespan. Importantly, DR suppresses reproductive rate, also in the presence of food odour, even when total reproduction is unaffected. We present a new evolutionary model that demonstrates that the DR‐driven lifespan extension can evolve via a route that does not require resource re‐allocation or other trade‐offs (Figure 1). Instead, the model assumes that DR suppresses reproduction, and this leads to enhanced selection for long life.

If genes are expressed differently in DR and non‐DR environments, then this change in natural selection can be sufficient for the evolution of increased somatic maintenance in DR environments. Such differential gene expression is commonly reported in genomic studies of DR (Weindruch et al. 2001; Han and Hickey 2005; Sultanova et al. 2025). This parsimonious model is rooted in standard evolutionary theory, and reconciles empirical findings presented here with the previous studies. This model suggests that lifespan extension under DR evolves under a broader range of conditions than has been previously considered, namely a relaxation of the previous assumption that individual organisms experience both environments during their lifetime. Furthermore, because trade‐offs are unnecessary for this model to work, the DR response can evolve via germline mutation accumulation consistent with the Mutation Accumulation model of ageing (Medawar 1952). Importantly, this model is in line with experimental evolution studies suggesting that the evolution of fertility and lifespan under DR can be uncoupled (Zajitschek et al. 2016; Zajitschek et al. 2019).

While this DR‐driven longevity via mutation accumulation model (DRM) does not require any trade‐offs for increased somatic maintenance under DR to evolve, it does not preclude them. Where trade‐offs between reproduction and survival exist, whether longer lifespan under DR should evolve depends upon how realised costs of reproduction differ between DR and *ad libitum* conditions and on the pattern of age‐specific fertility.

### 3.1 To Reproduce, or Not to Reproduce

Disentangling the physiological versus selection‐based effects of resource limitation on reproduction was possible in our experiment by engaging both gustatory and olfactory responses in *C. elegans*. By providing the odour of the food, but without actual food available to eat, only the nematodes' olfactory responses were engaged, which was enough to induce reproduction, albeit at a lower rate than fully fed individuals. This indicates that although DR individuals carry enough resources to produce some offspring (whether this is through recycling extraneous cellular and protein materials or reserves), they reproduce less when they sense that their environment is poor. Even when reproduction is induced in the presence of food odour without access to food, there does not appear to be any trade‐off between reproduction and somatic maintenance. Another study found that inducing reproduction in the absence of food did not extend lifespan to the same degree as DR alone, indicating a trade‐off (Ivimey‐Cook et al. 2021). However, their treatment was 48 h of starvation compared to our 24‐h treatment, highlighting that the line between DR and malnutrition can be difficult to discern.

Our results also contradict the notion that the DR response is solely a laboratory artefact. It is known that the *E. coli* fed to laboratory *C. elegans* is damaging, especially at older ages, and shortens lifespan (Walker et al. 2005), but our transient treatment ensures that the effects on nematodes subjected to DR cannot be due to avoidance of such effects. Furthermore, our study design indicates that the delay in reproduction is partially decision‐based, rather than solely a consequence of limited resource availability. Resource availability plays an integral role in determining the life cycle of *C. elegans* during development (Golden and Riddle 1984), and it is likely that selection has also acted on response to food availability during reproductive stages. Some suggest that the DR response encompasses much more than response to nutrient availability, which is likely only a part of a response to conditions expected to coincide with resource scarcity in nature, such as heatwaves or drought (Regan et al. 2020). This is consistent with our results and those of others who show that nutrient restriction improves resistance to stress (Smith et al. 2008). Improved stress resistance may explain the results we see in our semi‐natural populations following DR treatment with or without food odour. While worms from both treatments reproduce more slowly, improved resistance to stress may compensate for this compared to our control treatments, allowing post‐DR populations to grow at similar rates and DR with food odour populations to grow faster.

Our results and model demonstrate that previous trade‐off‐based models are neither sufficient nor necessary to explain lifespan extension under DR in the general case. We show that germline mutation accumulation could lead to the evolution of shorter lifespans in environments where nutrients are plentiful. With the plethora of conflicting results across species, genotypic backgrounds, and laboratory techniques throughout the field, it is no surprise that both the evolution and mechanisms of DR remain hotly debated. We suggest that our model provides a general and parsimonious explanation for the evolution of increased somatic maintenance and lifespan extension under DR. Importantly, this model can be used to predict the evolution of longer lifespan under any environmental stress that suppresses fertility and, therefore, changes selection gradients for adult survival in any organism.

### 3.2 Broad Implications of the Model

While we focused here on the reasons behind DR‐driven lifespan extension, it is important to note that this model is broadly applicable to any kind of environmental stress that reduces fertility. The evolution of hormesis, defined here as non‐lethal stress that increases somatic maintenance and lifespan, is a long‐standing problem in biology. The model presented here suggests that any kind of non‐lethal stress that reduces reproduction will result in selection favouring increased survival, and therefore, increased somatic maintenance. This process suggests that hormesis is expected to evolve under a very broad range of conditions, because reduced fertility often accompanies non‐lethal stress. For example, elegant work on germline‐soma interactions in *C. elegans* showed that when the germline is damaged by the environmental stress and needs time to recover, it upregulates systemic stress response in the somatic cells resulting in increased survival and stress resistance (Ermolaeva et al. 2013). This example fits perfectly with the model presented here, because extrinsic damage to germline reduces fertility and changes selection gradients for survival. The model suggests that selection on upregulation of innate immune response and increased somatic endurance when germline integrity is temporarily compromised is stronger than when the germline is intact, and fertility is high.

## 4 Methods

### 4.1 Experimental Model

N2 wild‐type *Caenorhabditis elegans* nematodes were obtained from the *Caenorhabditis* Genetics Centre (funded by NIH Office of Research Infrastructure Programs–P40 OD010440). Populations maintained in −70°C were thawed, then bleached using a solution of NaOCl and NaOH to rid the population of fungal and bacterial contamination. To prevent effects of freezing from impacting experimental work, populations were maintained for at least two generations on 90 mm Nematode Growth Medium (NGM) agar plates made with 10 mg ml^−1^ of Nystatin and 100 mg ml^−1^ of ampicillin at 20°C. Plates were seeded with *Escherichia coli* OP50‐1 (pUC4 K), obtained from J. Ewbank at the Centre d'Immunologie de Marseille‐Luminy, France to feed the populations. Short‐duration timed egg lays, where peak reproductive‐age adults were allowed to lay eggs on a plate for a maximum of 2 h then removed, were used to produce age‐synchronised populations.

### 4.2 Experimental Design

The experiments described here consisted of four treatments: (1) *ad libitum* food (F); (2) *ad libitum* food plus food odour (F + O); (3) dietary restriction through removal of food for 24 h (DR); and (4) dietary restriction through removal of food for 24 h with exposure to food odour (DR + O). The full bacterial removal is a DR method that has been successfully used by different laboratories (Smith et al. 2008; Sutphin and Kaeberlein 2008; Lee et al. 2006), including ours (Ivimey‐Cook et al. 2021). It produces a classic DR response with increased lifespan and reduced reproduction, and prevents the animals from increasing their food intake, as can happen when food dilution is used to induce DR. The control treatment (F) consisted of NGM agar plates seeded with a lawn of *E. coli*. DR plates were made without peptone, which is included in NGM agar plates to allow bacterial growth, and were left unseeded. DR and fully fed plates with food odour contained two agar layers, both with a bottom layer of NGM agar seeded with *E. coli* that was allowed to grow overnight. Fully fed plates then had a second layer identical to the bottom layer. DR with odour plates had a second layer made of NGM agar without peptone and were left unseeded. Bacterial incubation times were standardised across treatments.

For all experiments described here, age‐synchronised populations of nematodes were produced with timed egg lays. Upon reaching late L4 stage, nematodes were placed on their treatment of fully fed (F), fully fed with food odour (F + O), dietary restriction (DR), or DR with food odour (DR + O) for a 24‐h period. Excess individuals were placed onto DR treatments due to high risk of unfed individuals crawling up the side of the plastic plates and dying. When more than the desired number of individuals remained after treatment, the necessary number of individuals were randomly selected for use in the assay. Unless otherwise explicitly stated, all assays were performed in unmated hermaphrodites.

### 4.3 Lifespan and Reproduction Assays

First, the effects of DR with and without food odour on reproduction and longevity were tested in the laboratory, along with fully fed controls and fully fed with food odour positive controls. Individuals were kept on their own 35 mm NGM plates for reproduction assays, and lifespan assays consisted of 35 mm NGM plates containing 10 individuals. To determine daily survival, observations of touch‐response were used.

To test reproductive effects of DR with and without food odour, individuals from all treatments were transferred to new plates every 24 h. Eggs obtained each day were reared for 2 days, then killed and counted. Assays were fully blinded following the initial treatment day. Reproduction assays continued until approximately 75% of individuals stopped reproducing for at least 1 day.

### 4.4 Mated Reproduction Assay

To identify what role sperm limitation in hermaphrodites may play in reduced reproduction following DR, non‐experimental males were introduced to hermaphrodites for 24 h. Reproduction after mating was measured using the methods described above. Mating took place immediately following treatment, on day two of adulthood. Mating had to be performed after treatment to avoid treatment effects on males impacting the experiment. Each reproductive assay plate held 1 hermaphrodite and two males for 24‐h. The males were removed upon transfer to day three reproductive assay plates, and the assay resumed as described above.

### 4.5 Body and Egg Size Assays

Following treatment, on days two and four of adulthood, hermaphrodites were placed on fresh plates and immediately photographed using a Leica MC170 HD camera attached to a Leice M165 C light microscope. The unmated individuals were then allowed to lay eggs for 2 h. After that, up to three eggs were randomly chosen to take images of. Days two and four were chosen according to the reproductive peaks of fully fed worms (day two) and starved worms (day four). Surface area measurements were taken in Imagej (Schneider et al. 2012) by turning colour images into binary images, filling in transparent areas in individuals not captured by the automated binary tool in ImageJ, and measuring the black areas. The same method was used to measure eggs.

### 4.6 Heat Shock Assay

The heat shock assay was performed similarly to a lifespan experiment. Following exposure to their respective treatments, nematodes were placed onto fresh seeded plates in groups of 10, including only unmated hermaphrodites. The plates were then placed in a 37°C incubator and checked hourly for response to touch. As heat shock can cause immobility in nematodes, those assumed dead were kept on the plate in a designated location to confirm mortality at subsequent checks. Heat shock lasted 9 h, after which surviving nematodes were kept at 20°C and monitored for survival over the following days until all individuals died.

### 4.7 Outdoor Assay

Outdoor populations were housed in organic, peat‐free compost in 250‐ml conical tubes. The tubes had holes in the bottom to allow for drainage in the case of excess rain. The tubes were suspended above a bath of heavily salted water to kill any nematodes that escape through those holes to prevent environmental contamination. Compost was autoclaved no more than 7 days before the start of the experiment, with OP50 *E. coli* (susceptible to antibiotics) being reintroduced to the compost as a food source for the populations. The OP50 was maintained in LB broth at 4°C after growing at 37°C for 18 h. The compost was completely saturated in OP50‐inoculated LB broth (60 mL) and allowed to grow at room temperature for 24 h before nematodes were introduced.

All treatments were given on 90 mm agar plates for this experiment, due to the high number of nematodes required. Following treatment, 50 individuals were collected from a given treatment and placed on a clean 35‐mm agar plate, then rinsed into a tube of compost using OP50 bacterial broth. Outdoor populations were fed weekly with 25 mL of OP50.

Outdoor populations were sampled weekly over a three‐week period. Each week, a 5‐ml sample of compost was retrieved from each tube, after mixing the top half of the compost in each conical tube. Samples were cleaned with osmosis purified (RO) water to remove small particles by dissolving the compost in RO water at a total volume of 10 mL and centrifuging at 1800 g for 2 min, then pouring out the supernatant. Following this cleaning, the compost was then dissolved in a MgSO_4_ solution with a specific density of 1.15 (at which nematodes will be suspended) at a total volume of 10 mL and again centrifuged at 1800 g for 2 min. The supernatant was then carefully mixed with a pipette, then 0.1‐ml samples of the supernatant were pipetted onto 35‐mm agar plates and allowed to dry for future counting. For each population, six technical replicate samples were taken.

### 4.8 Statistical Analysis

All analyses were performed in R version 4.4.1 (R Core Team 2022). Model diagnostics were performed with the DHARMa package (Hartig et al. 2024). Where overdispersion was present in generalised linear mixed models, observation level random effects were included to control for it. Model selection was decided by choosing models with the lowest Akaike information criterion (AIC) based on error structures, zero‐inflation parameters, and dispersion parameters. Bootstrap‐coupled estimation plots were produced with the *dabestr* package (Ho et al. 2019), and all other plots were created using *ggplot2* (Wickham et al. 2024).

Survival assays were analysed by using cox proportional hazards models with mixed effects from the *coxme* (Therneau 2024) and *survival* (Therneau et al. 2024) packages. These models fit Treatment as a fixed effect, and Plate ID as a random intercept. This model was performed with and without matricides. Heat shock survival data was also analysed using these models with the same parameters.

Age‐specific reproduction was analysed using zero‐inflated negative binomial generalised linear mixed models (GLMMs) with the *glmmTMB* package (Brooks et al. 2019) with observation level random effects, including interacting fixed factors of Treatment, Day^2^, and Day, and individual ID as a random intercept to account for repeated measures of individuals' reproductive output over time. The full model for unmated age‐specific reproduction is as follows: No_offspring ~ Treatment * poly(Day, 2) + (1|ID), zi = ~Day, disp = ~Day. For mated reproduction, the final model is as follows: No_offspring ~ Treatment * poly(Day, 2) + (1|ID), zi = ~ poly(Day,2), disp = ~Treatment*Day^2^.

The effects of dietary treatment on lifetime reproductive success and individual fitness and body size were analysed using linear models with Gaussian error structures, and to identify differences between treatments, the *emmeans* package (Lenth 2024) was used to perform pairwise comparisons. The effect of dietary treatment on egg size was analysed using linear mixed effects models with Gaussian error structures with Treatment as a fixed factor and ID as a random intercept to account for repeated measures of technical replicates per individual.

Population growth data was analysed using zero‐inflated negative binomial GLMMs with the *glmmTMB* package. Treatment, Day^2^, Day and Block were fit as fixed factors, with all interactions in the initial model. The final model is as follows: Population index ~ Treatment * poly(Day, 2) * Block. Technical replicate nested within Population was included as a random intercept to account for repeated measures within populations across replicates and time. To account for problems with overdispersion and zero inflation, formulas were fit for each. The final model includes a ziformula = ~ Day + Block.

## Author Contributions

Conceptualization: S.D.I., S.I., J.M., A.A.M. Formal analysis: S.D.I., J.M. Funding acquisition: S.I., A.A.M. Investigation: S.D.I., A.K., J.M., A.A.M. Methodology: S.D.I., J.M., A.A.M. Supervision: S.I., J.M., A.A.M. Visualisation: S.D.I. Writing – original draft: S.D.I., J.M., A.A.M. Writing – review and editing: S.D.I., A.K., S.I., J.M., A.A.M.

## Funding

European Research Council consolidator grant SELECTHAPLOID 101001341 awarded to S.I. Natural Environment Research Council grant NE/W001020/1 awarded to A.A.M.

## Conflicts of Interest

The authors declare no conflicts of interest.

## Supporting information


**Table S1:** Cox mixed effects model summary for nematode survival data following treatments of *ad libitum* food (F), *ad libitum* food plus odour (F + O), dietary restriction (24‐h starvation; DR), or dietary restriction plus odour (DR + O). Model was performed on data including and excluding matricides as deaths, and results from data censoring matricides is used for interpretation and post hoc analysis.
**Table S2:** Zero‐inflated negative binomial generalised linear mixed effects model results for analysis of age‐specific reproduction over 8 days in nematodes following treatments of *ad libitum* food (F), *ad libitum* food plus odour (F + O), dietary restriction (24‐h starvation; DR), or dietary restriction plus odour (DR + O).
**Table S3:** Linear model output from analysis of lifetime reproductive success (LRS, total number of offspring produced) in nematodes following treatments of *ad libitum* food (F), *ad libitum* food plus odour (F + O), dietary restriction (24‐h starvation; DR), or dietary restriction plus odour (DR + O).
**Table S4:** Linear mixed effects model output for analysis of egg size on Days 2 and 4 of adulthood in nematodes following Day 1 treatment of *ad libitum* food (F), *ad libitum* food plus odour (F + O), dietary restriction (24‐h starvation; DR), or dietary restriction plus odour (DR + O).
**Table S5:** Zero‐inflated negative binomial generalised linear mixed effects model results for analysis of age‐specific reproduction over 10 days in mated nematodes following treatments of *ad libitum* food (F), *ad libitum* food plus odour (F + O), dietary restriction (24‐h starvation; DR), or dietary restriction plus odour (DR + O).
**Table S6:** Linear model output from analysis of lifetime reproductive success (LRS, total number of offspring produced) in mated nematodes following treatments of *ad libitum* food (F), *ad libitum* food plus odour (F + O), dietary restriction (24‐h starvation; DR), or dietary restriction plus odour (DR + O).
**Table S7:** Linear model output for analysis of adult nematode body size on Days 2 and 4 of adulthood and growth between Days 2 and 4 following treatment on Day 1 of adulthood of *ad libitum* food (F), *ad libitum* food plus odour (F + O), dietary restriction (24‐h starvation; DR), or dietary restriction plus odour (DR + O).
**Table S8:** Post hoc analysis using Tukey method of linear model analysis of growth between Days 2 and 4 of adulthood in nematodes following treatments of *ad libitum* food (F), *ad libitum* food plus odour (F + O), dietary restriction (24‐h starvation; DR), or dietary restriction plus odour (DR + O).
**Table S9:** Cox mixed effects model summary for nematode heat shock (37°C) survival data following mating after treatments of *ad libitum* food (F), *ad libitum* food plus odour (F + O), dietary restriction (24‐h starvation; DR), or dietary restriction plus odour (DR + O). Time points included hours 1–9 of heat shock treatment, and each subsequent time point is a day following heat shock treatment.
**Table S10:** Zero‐inflated negative binomial GLMM analysis of population growth in semi‐natural conditions following day one adulthood treatment of *ad libitum* (F), *ad libitum* + odour (F + O), dietary restriction (DR), or dietary restriction+ odour (DR + O).
**Table S11:** Type III Anova output for zero‐inflated negative binomial generalised linear mixed model (Table S11) for analysis of population growth in semi‐natural conditions over 21 days following treatment on Day 1 of adulthood of *ad libitum* food (F), *ad libitum* food plus odour (F + O), dietary restriction (24‐h starvation; DR), or dietary restriction plus odour (DR + O).

## Data Availability

All code and data used for this study are available via GitHub at https://github.com/irishsar92/DR_MAT.
