# Supplementary material for: Mutation Accumulation Underpins Evolution of Lifespan Extension by Dietary Restriction
Source: Aging Cell. 2026 Nov 5;25(11):e70808. doi: 10.1111/acel.70808 (PMC13740021; doi:10.1111/acel.70808)
Supplement: Supplementary file 1 [file ACEL-25-e70808-s001.docx]

Supplementary Materials for

**Mutation accumulation underpins evolution of lifespan extension by dietary restriction**

Sara D. Irish^*^, Annabel Kimberley, Simone Immler, Jacob Moorad,

Alexei A. Maklakov

*Corresponding author email: s.irish@uea.ac.uk

**This file contains:**

**Tables S1 to S11**

**Table S1:** Cox mixed effects model summary for nematode survival data following treatments of *ad libitum* food (F), *ad libitum* food plus odour (F+O), dietary restriction (24-hour starvation; DR), or dietary restriction plus odour (DR+O). Model was performed on data including and excluding matricides as deaths, and results from data censoring matricides is used for interpretation and post-hoc analysis.

| **Matricides included** | | | | | | | | | | |
| --- | --- | --- | --- | --- | --- | --- | --- | --- | --- | --- |
|  | *Coefficient* | | *Exp(Coefficient)* | | *Std. Error* | | *z* | | *p* | |
| DR | -1.0014 | | 0.3674 | | 0.1755 | | -5.71 | | <0.0001 | |
| DR+O | -1.0254 | | 0.3587 | | 0.1775 | | -5.78 | | <0.0001 | |
| F+O | -0.1141 | | 0.8922 | | 0.1689 | | -0.68 | | 0.499 | |
| Random effects: Plate.ID | | | Variance: 0.0282 | | | | Std. Deviation: 0.1682 | | | |
|  |  | |  | |  | |  | |  | |
| **Matricides censored** | | | | | | | | | | |
| DR | -1.0494 | | 0.3502 | | 0.1766 | | -5.94 | | <0.0001 | |
| DR+O | -1.0745 | | 0.3415 | | 0.1788 | | -6.01 | | <0.0001 | |
| F+O | -0.9579 | | 0.9087 | | 0.1690 | | -0.57 | | 0.571 | |
| Random effects: Plate.ID | | | Variance: 0.0244 | | | | Std. Deviation: 0.1561 | | | |
|  | | |  | | | |  | | | |
| **Tukey post-hoc comparisons (Matricides censored)** | | | | | | | | | | |
|  | | *Estimate* | | *Std. Error* | | *z* | | *p* | |  |
| F – DR | | 1.094 | | 0.177 | | 5.943 | | <0.0001 | |  |
| F – DR+O | | 1.075 | | 0.179 | | 6.009 | | <0.0001 | |  |
| F – F+O | | 0.096 | | 0.169 | | 0.567 | | 0.942 | |  |
| DR – DR+O | | 0.025 | | 0.173 | | 0.145 | | 0.999 | |  |
| DR – F+O | | -0.954 | | 0.176 | | -5.403 | | <0.0001 | |  |
| DR+O – F+O | | -0.979 | | 0.179 | | -5.476 | | <0.0001 | |  |
|  | | |  | | | |  | | | |

**Table S2:** Zero-inflated negative binomial generalised linear mixed effects model results for analysis of age-specific reproduction over eight days in nematodes following treatments of *ad libitum* food (F), *ad libitum* food plus odour (F+O), dietary restriction (24-hour starvation; DR), or dietary restriction plus odour (DR+O).

| **Model: No_offspring ~ Treatment*Day + Treatment*Day^2 + (1\|ID), zi = ~ Treatment** | | | | | | | | | |
| --- | --- | --- | --- | --- | --- | --- | --- | --- | --- |
|  | *Estimate* | | *Std. Error* | | *z-value* | | | *p-value* |  |
| (Intercept) | 3.928 | | 0.267 | | 14.705 | | | <0.0001 |  |
| F+O | -0.616 | | 0.394 | | -1.563 | | | 0.118 |  |
| DR | -4.826 | | 0.355 | | -13.579 | | | <0.0001 |  |
| DR+O | -2.179 | | 0.355 | | -6.133 | | | <0.0001 |  |
| Day | 0.719 | | 0.162 | | 4.436 | | | <0.0001 |  |
| Day^2^ | -0.214 | | 0.021 | | -9.984 | | | <0.0001 |  |
| F+O: Day | 0.543 | | 0.246 | | 2.204 | | | 0.028 |  |
| DR: Day | 2.045 | | 0.203 | | 10.052 | | | <0.0001 |  |
| DR+O: Day | 1.094 | | 0.211 | | 5.181 | | | <0.0001 |  |
| F+O: Day^2^ | -0.101 | | 0.034 | | -2.954 | | | 0.003 |  |
| DR: Day^2^ | -0.143 | | 0.026 | | -5.536 | | | <0.0001 |  |
| DR+O: Day^2^ | -0.096 | | 0.028 | | -3.455 | | | 0.0006 |  |
|  |  | |  | |  | | |  |  |
| **Zero-inflation formula = ~ Treatment** | | | | | | | | | |
| (Intercept) | -2.906 | | 0.504 | | -5.759 | | | <0.0001 |  |
| F+O | -0.752 | | 1.006 | | -0.748 | | | 0.455 |  |
| DR | -4.102 | | 14.182 | | -0.289 | | | 0.772 |  |
| DR+O | -1.299 | | 1.078 | | -1.205 | | | 0.228 |  |
|  |  | |  | |  | | |  |  |
| Random effects: ID | | | Variance: 0.130 | | | Std. Deviation: 0.360 | | | |
|  | | |  | | |  | | | |
| **Type III Anova** | | |  | | |  | | | |
|  | | $\chi^{2}$ | | *df* | | | *p* | | |
| (Intercept) | | 216.250 | | 1 | | | <0.0001 | | |
| Treatment | | 220.297 | | 3 | | | <0.0001 | | |
| Day | | 19.678 | | 1 | | | <0.0001 | | |
| Day^2^ | | 99.683 | | 1 | | | <0.0001 | | |
| Treatment*Day | | 112.655 | | 3 | | | <0.0001 | | |
| Treatment* Day^2^ | | 30.688 | | 3 | | | <0.0001 | | |

**Table S3:** Linear model output from analysis of lifetime reproductive success (LRS, total number of offspring produced) in nematodes following treatments of *ad libitum* food (F), *ad libitum* food plus odour (F+O), dietary restriction (24-hour starvation; DR), or dietary restriction plus odour (DR+O).

| **Model: LRS ~ Treatment:** R^2^ = 0.111, F_3,122_ = 6.219, p = 0.0006 | | | | |
| --- | --- | --- | --- | --- |
|  | *Estimate* | *Std. Error* | *t-value* | *p-value* |
| (Intercept) | 302.935 | 7.136 | 42.453 | <0.0001 |
| F+O | 5.765 | 10.175 | 0.567 | 0.572 |
| DR | -30.087 | 9.937 | -3.028 | 0.003 |
| DR+O | -24.217 | 10.012 | -2.419 | 0.017 |
|  |  |  |  |  |

**Table S4:** Linear mixed effects model output for analysis of egg size on Days 2 and 4 of adulthood in nematodes following Day 1 treatment of *ad libitum* food (F), *ad libitum* food plus odour (F+O), dietary restriction (24-hour starvation; DR), or dietary restriction plus odour (DR+O).

| **Model: Day 2 Egg Size ~ Treatment + (1\|ID):** *Χ^2^=* 27.262, df = 3, *p* < 0.0001 | | | | | |
| --- | --- | --- | --- | --- | --- |
|  | *Estimate* | *Std. Error* | *df* | *t-value* | *p-value* |
| (Intercept) | 1.677*10^-3^ | 2.813*10^-3^ | 91.77 | 59.616 | <0.0001 |
| F+O | -2.609*10^-5^ | 3.974*10^-3^ | 91.47 | -0.657 | 0.513 |
| DR | -1.569*10^-4^ | 3.981*10^-3^ | 92.07 | -3.941 | <0.001 |
| DR+O | -1.606*10^-4^ | 3.978*10^-3^ | 91.77 | -4.037 | <0.001 |
| Random effects: ID | | 1.545*10^-8^ | Std. Deviation | | 1.243*10^-4^ |
| Residual variance: | | 1.041*10^-8^ | Std. Deviation | | 1.020*10^-4^ |
|  |  |  |  |  |  |
| **Model: Day 4 Egg Size ~ Treatment + (1\|ID):** *Χ^2^=* 22.307, df = 3, *p* < 0.0001 | | | | | |
| (Intercept) | 1.608*10^-3^ | 2.312*10^-5^ | 99.88 | 69.540 | <0.0001 |
| F+O | 5.186*10^-5^ | 3.288*10^-5^ | 94.3 | 1.577 | 0.118 |
| DR | -8.073*10^-5^ | 3.187*10^-5^ | 105.50 | -2.533 | 0.013 |
| DR+O | -7.125*10^-5^ | 3.248*10^-5^ | 93.71 | -2.194 | 0.031 |
| Random effects: ID | | 9.999*10^-9^ | Std. Deviation | | 1.000*10^-4^ |
| Residual variance: | | 9.015*10^-9^ | Std. Deviation | | 9.495*10^-5^ |

**Table S5:** Zero-inflated negative binomial generalised linear mixed effects model results for analysis of age-specific reproduction over ten days in mated nematodes following treatments of *ad libitum* food (F), *ad libitum* food plus odour (F+O), dietary restriction (24-hour starvation; DR), or dietary restriction plus odour (DR+O).

| **Model: No_offspring ~ Treatment * poly(Day, 2) + (1\|ID), zi = ~ Day** | | | | | | | | | | |
| --- | --- | --- | --- | --- | --- | --- | --- | --- | --- | --- |
|  | | *Estimate* | | *Std. Error* | | *z-value* | | | *p-value* |  |
| (Intercept) | | 4.597 | | 0.172 | | 26.777 | | | <0.0001 |  |
| F+O | | -0.317 | | 0.236 | | -1.340 | | | 0.180 |  |
| DR | | -2.531 | | 0.269 | | -9.421 | | | <0.0001 |  |
| DR+O | | -1.127 | | 0.248 | | -4.550 | | | <0.0001 |  |
| Day | | 0.055 | | 0.073 | | 0.761 | | | 0.447 |  |
| Day^2^ | | -0.028 | | 0.007 | | -4.331 | | | 0.0001 |  |
| F+O: Day | | 0.190 | | 0.099 | | 1.929 | | | 0.054 |  |
| DR: Day | | 0.849 | | 0.108 | | 7.861 | | | <0.0001 |  |
| DR+O: Day | | 0.421 | | 0.102 | | 4.144 | | | 0.0003 |  |
| F+O: Day^2^ | | -0.018 | | 0.009 | | -2.065 | | | 0.039 |  |
| DR: Day^2^ | | -0.062 | | 0.009 | | -6.524 | | | <0.0001 |  |
| DR+O: Day^2^ | | -0.031 | | 0.009 | | -3.413 | | | 0.0006 |  |
|  |  |  |  |  |  |  |  |  |  |  |
| **Zero-inflation formula = ~Treatment*Day** | | | | | | | | | | |
| (Intercept) | | -7.525 | | 1.408 | | -5.344 | | | <0.0001 |  |
| F+O | | -4.333 | | 3.330 | | -1.301 | | | 0.193 |  |
| DR | | 3.971 | | 1.539 | | 2.580 | | | 0.010 |  |
| DR+O | | 0.965 | | 1.899 | | 0.508 | | | 0.611 |  |
| Day^2^ | | 0.746 | | 0.163 | | 4.574 | | | <0.0001 |  |
| F+O: Day^2^ | | 0.382 | | 0.364 | | 1.049 | | | 0.294 |  |
| DR: Day^2^ | | -0.470 | | 0.183 | | -2.569 | | | 0.010 |  |
| DR+O: Day^2^ | | -0.161 | | 0.221 | | -0.730 | | | 0.465 |  |
|  | |  | |  | |  | | |  |  |
| Random effects: ID | | | | Variance: 0.036 | | | Std. Deviation: 0.189 | | | |
|  | | | | | | | | | | |
| Type III Anova | | | | | | | | | | |
|  | | | $\chi^{2}$ | | *df* | | | *p* | | |
| (Intercept) | | | 717.008 | | 1 | | | <0.0001 | | |
| Treatment | | | 103.841 | | 3 | | | <0.0001 | | |
| Day | | | 0.579 | | 1 | | | 0.447 | | |
| Day^2^ | | | 18.755 | | 1 | | | 0.0001 | | |
| Treatment*Day | | | 68.701 | | 3 | | | <0.0001 | | |
| Treatment*Day^2^ | | | 45.304 | | 3 | | | <0.0001 | | |

**Table S6:** Linear model output from analysis of lifetime reproductive success (LRS, total number of offspring produced) in mated nematodes following treatments of *ad libitum* food (F), *ad libitum* food plus odour (F+O), dietary restriction (24-hour starvation; DR), or dietary restriction plus odour (DR+O).

| **Model: Mated LRS ~ Treatment:** R^2^ = 0.176, F_3,67_ = 5.997, *p* = 0.001 | | | | |
| --- | --- | --- | --- | --- |
|  | *Estimate* | *Std. Error* | *t-value* | *p-value* |
| (Intercept) | 568.45 | 31.01 | 18.333 | <0.0001 |
| F+O | 42.79 | 43.33 | 0.988 | 0.327 |
| DR | -155.09 | 48.32 | -3.210 | 0.002 |
| DR+O | -26.89 | 46.51 | -0.578 | 0.565 |
|  |  |  |  |  |

**Table S7:** Linear model output for analysis of adult nematode body size on Days 2 and 4 of adulthood and growth between Days 2 and 4 following treatment on Day 1 of adulthood of *ad libitum* food (F), *ad libitum* food plus odour (F+O), dietary restriction (24-hour starvation; DR), or dietary restriction plus odour (DR+O).

| **Model: Day 2 Body Size ~ Treatment:** R^2^ = 0.929, F_3, 92_ = 415, *p <* 0.0001 | | | | |
| --- | --- | --- | --- | --- |
|  | *Estimate* | *Std. Error* | *t-value* | *p-value* |
| (Intercept) | 0.0581 | 0.0007 | 84.429 | <0.0001 |
| F+O | -0.0024 | 0.0010 | -2.505 | 0.014 |
| DR | -0.0246 | 0.0010 | -25.517 | <0.0001 |
| DR+O | -0.0253 | 0.0010 | -26.232 | <0.0001 |
|  |  |  |  |  |
| **Model: Day 4 Body Size ~ Treatment:** R^2^ = 0.744, F_3, 92_ = 92.77, *p <* 0.0001 | | | | |
| (Intercept) | 0.0655 | 0.0010 | 63.584 | <0.0001 |
| F+O | 0.0007 | 0.0015 | 0.502 | 0.617 |
| DR | -0.0166 | 0.0014 | -11.538 | <0.0001 |
| DR+O | -0.0134 | 0.0014 | -9.318 | <0.0001 |
|  |  |  |  |  |
| **Model: (D4 Size–D2 Size) ~ Treatment:** R^2^ = 0.503, F_3, 92_ = 33, *p <* 0.0001 | | | | |
| (Intercept) | 0.0074 | 0.0010 | 7.422 | <0.0001 |
| F+O | 0.0032 | 0.0014 | 2.232 | 0.028 |
| DR | 0.0079 | 0.0014 | 5.643 | <0.0001 |
| DR+O | 0.0118 | 0.0014 | 8.413 | <0.0001 |

**Table S8:** Post-hoc analysis using Tukey method of linear model analysis of growth between Days 2 and 4 of adulthood in nematodes following treatments of *ad libitum* food (F), *ad libitum* food plus odour (F+O), dietary restriction (24-hour starvation; DR), or dietary restriction plus odour (DR+O).

| *Contrast* | *Estimate* | *SE* | *t-ratio* | *p-value* |
| --- | --- | --- | --- | --- |
| F * F+O | -0.0032 | 0.0014 | -2.232 | 0.122 |
| F * DR | -0.0079 | 0.0014 | -5.643 | <0.0001 |
| F * DR+O | -0.0118 | 0.0014 | -8.413 | <0.0001 |
| F+O * DR | -0.0048 | 0.0014 | -3.388 | 0.006 |
| F+O * DR+O | -0.0087 | 0.0014 | -6.158 | <0.0001 |
| DR * DR+O | -0.0039 | 0.0014 | -2.799 | 0.031 |
| df = 94 for all comparisons | | | | |

**Table S9:** Cox mixed effects model summary for nematode heat shock (37˚C) survival data following mating after treatments of *ad libitum* food (F), *ad libitum* food plus odour (F+O), dietary restriction (24-hour starvation; DR), or dietary restriction plus odour (DR+O). Time points included hours 1-9 of heat shock treatment, and each subsequent time point is a day following heat shock treatment.

|  | *Coefficient* | *Exp(Coefficient)* | *Std. Error* | *z* | *p* |
| --- | --- | --- | --- | --- | --- |
| F+O | 0.0923 | 1.097 | 0.144 | 0.64 | 0.522 |
| DR | -3.181 | 0.042 | 0.211 | -15.10 | <0.0001 |
| DR+O | -3.436 | 0.032 | 0.214 | -16.03 | <0.0001 |
| Random effects: Plate.ID | | Variance: 8.54*10^-5^ | | Std. Deviation: 0.0092 | |
|  |  |  |  |  |  |

**Table S10:** Zero-inflated negative binomial GLMM analysis of population growth in semi-natural conditions following day one adulthood treatment of *ad libitum* (F), *ad libitum*+odour (F+O), dietary restriction (DR), or dietary restriction+ odour (DR+O).

| **Population index ~ Treatment *poly(Day, 2) * Block + (1\|Population/Replicate)** | | | | | |
| --- | --- | --- | --- | --- | --- |
|  | *Estimate* | *Std. Error* | *z-value* | | *p-value* |
| (Intercept) | 3.546 | 0.253 | 14.033 | | <0.0001 |
| F+O | -0.006 | 0.349 | -0.017 | | 0.9861 |
| DR | -0.108 | 0.349 | -0.309 | | 0.7573 |
| DR+O | 0.213 | 0.341 | 0.624 | | 0.5325 |
| Day | 5.118 | 2.329 | 2.198 | | 0.0280 |
| Day^2^ | -26.519 | 2.138 | -12.405 | | <0.0001 |
| Block 2 | -2.092 | 0.352 | -5.950 | | <0.0001 |
| Block 3 | 1.101 | 0.382 | 2.883 | | 0.0039 |
| F+O*Day | 16.290 | 3.365 | 4.841 | | <0.0001 |
| DR*Day | 22.880 | 3.333 | 6.865 | | <0.0001 |
| DR+O*Day | 16.501 | 3.247 | 5.082 | | <0.0001 |
| F+O*Day^2^ | 3.894 | 3.131 | 1.244 | | 0.2137 |
| DR*Day^2^ | -3.877 | 3.086 | -1.256 | | 0.2090 |
| DR+O*Day^2^ | -2.036 | 2.909 | -0.700 | | 0.4841 |
| F+O*Block 2 | 0.254 | 0.490 | 0.519 | | 0.6035 |
| DR*Block 2 | 0.489 | 0.490 | 0.997 | | 0.3187 |
| DR+O*Block 2 | 0.180 | 0.485 | 0.371 | | 0.7108 |
| F+O*Block 3 | 0.049 | 0.534 | 0.091 | | 0.9276 |
| DR*Block 3 | -1.007 | 0.526 | -1.914 | | 0.0556 |
| DR+O*Block 3 | -0.149 | 0.519 | -0.287 | | 0.7743 |
| Day*Block 2 | 13.867 | 4.039 | 3.433 | | 0.0006 |
| Day*Block 3 | -7.355 | 3.330 | -2.209 | | 0.0272 |
| Day^2^*Block 2 | 16.912 | 3.300 | 5.124 | | <0.0001 |
| Day^2^*Block 3 | 19.055 | 3.267 | 5.832 | | <0.0001 |
| F+O*Day*Block 2 | -37.531 | 5.389 | -6.964 | | <0.0001 |
| DR*Day*Block 2 | -30.723 | 5.442 | -5.645 | | <0.0001 |
| DR+O*Day*Block 2 | -21.088 | 5.431 | -3.883 | | 0.0001 |
| F+O*Day^2^*Block 2 | 3.504 | 4.685 | 0.748 | | 0.4545 |
| DR*Day^2^*Block 2 | 5.060 | 4.655 | 1.087 | | 0.2770 |
| DR+O*Day^2^*Block 2 | -10.305 | 4.534 | -2.273 | | 0.0230 |
| F+O*Day*Block 3 | -14.964 | 4.781 | -3.130 | | 0.0017 |
| DR*Day*Block 3 | -22.595 | 4.717 | -4.791 | | <0.0001 |
| DR+O*Day*Block 3 | -20.147 | 4.608 | -4.372 | | <0.0001 |
| F+O*Day^2^*Block 3 | -5.999 | 4.597 | -1.305 | | 0.1919 |
| DR*Day^2^*Block 3 | 7.721 | 4.559 | 1.694 | | 0.0903 |
| R+O*Day^2^*Block 3 | 7.378 | 4.379 | 1.685 | | 0.0920 |
| **Zero-inflation model: ~ Day^2^** | | | | | |
| (Intercept) | -0.197 | 0.510 | -0.386 | | 0.6997 |
| Day | -0.275 | 0.054 | -5.065 | | <0.0001 |
| Block 2 | -0.460 | 0.558 | -0.826 | | 0.4090 |
| Block 3 | -1.205 | 0.440 | -2.741 | | 0.0061 |
| *Random effects:* | | *Variance* | |  | *Std. Dev.* |
| Replicate: Population | | 1.782 * 10^-10^ | | 0.00001 | |
|  | Population | 0.542 | | 0.7445 | |

**Table S11:** Type III Anova output for zero-inflated negative binomial generalised linear mixed model (Table S11) for analysis of population growth in semi-natural conditions over 21 days following treatment on Day 1 of adulthood of *ad libitum* food (F), *ad libitum* food plus odour (F+O), dietary restriction (24-hour starvation; DR), or dietary restriction plus odour (DR+O).

|  | $\boldsymbol{\chi}^{\boldsymbol{2}}$ | **df** | ***p*-value** |
| --- | --- | --- | --- |
| (Intercept) | 4.351 | 1 | 0.0370 |
| Treatment | 10.819 | 3 | 0.0128 |
| Day | 153.965 | 1 | <0.0001 |
| Block | 91.333 | 2 | <0.0001 |
| Day^2^ | 153.756 | 1 | <0.0001 |
| Treatment*Day | 9.273 | 3 | 0.0259 |
| Treatment*Block | 39.736 | 6 | <0.0001 |
| Day*Block | 52.179 | 2 | <0.0001 |
| Treatment* Day^2^ | 6.613 | 3 | 0.0853 |
| Block* Day^2^ | 60.263 | 2 | <0.0001 |
| Treatment*Day*Block | 48.332 | 6 | <0.0001 |
| Treatment* Day^2^*Block | 40.688 | 6 | <0.0001 |
